# Method for Obtaining Committed Adult Mesenchymal Precursors from Skin and Lung Tissue

**DOI:** 10.1371/journal.pone.0053215

**Published:** 2012-12-31

**Authors:** Aurora Bernal, María Fernández, Laura M. Pérez, Nuria San Martín, Beatriz G. Gálvez

**Affiliations:** Centro Nacional de Investigaciones Cardiovasculares (CNIC), Madrid, Spain; Instituto Butantan, Brazil

## Abstract

**Aims:**

The present study reports an easy and efficient method for obtaining adult mesenchymal precursors from different adult mouse tissues.

**Materials and Methods:**

We describe the isolation and expansion of mesenchymal precursors from skin and lung by a non-enzymatic method. Skin and lung mesenchymal precursors isolated by a modified explant technique were characterized *in vitro* by defined morphology and by a specific gene expression profile and surface markers.

**Results and Conclusions:**

Our results show that these precursors express stem cell and mesenchymal surface markers as well as epithelial markers. However, they are negative for markers of endothelium, cardiac and skeletal muscle or adipose tissue, indicating that they have initiated commitment to the tissues from which were isolated. These precursors can migrate without any stimulus and in response to stimuli as SDF1, MCP1 and TNFα and can be differentiated into epithelial lineages. Based on the properties of these precursors from adult tissues, we propose their use as tools for regenerative biomedicine.

## Introduction

Regenerative medicine research has attracted increasing interest in recent years, but the clinical applications and outcomes require further analysis [1], [2]. To date, several kinds of stem cells have been evaluated as candidate tools for regenerative medicine, including embryonic and adult (mesenchymal and hematopoietic) stem cells [3]. Despite the fact that embryonic stem cells are highly efficient generators of many adult tissues, their therapeutic use has been limited due to their possible tumorigenicity, the need for immune suppression and the ethical limitations [4], [5]. However, the clinical application of these stem cells requires the development of minimally invasive standardized protocols for obtaining large numbers of homogeneous precursor cells.

[LOOSEST The presence of non-hematopoietic stem cells in bone marrow was first suggested by the observations of *Cohnheim* 130 years ago [6]. However, definitive evidence came with the method of *Friedenstein* et al. [7] for isolating mesenchymal stem cells (MSC), a multipotent cell population able to differentiate into osteoblasts, chondrocytes and adipocytes [8], [9]. MSCs have been isolated from amniotic fluid, periosteum, adipose tissue and fetal tissues by means of enzymatic protocols and from skeletal and cardiac tissues by the explant method [10], [11], [12], [13], [14], [15]. The fact that MSCs can be differentiated into several different cell types *in vitro*, together with their relative ease of expansion in culture and their immunologic characteristics, make MSCs and the related MSC-like cells promising sources of stem cells for tissue repair. More recently skin has attracted attention as is a highly accesible tissue and is considered a stem cells reservoir for numerous cell lineages [16], [17], [18], [19], [20], [21], [22]. In fact, it has been demonstrated that dermal and hair follicle precursors cells are able to differentiate into different lineages [16], [20], [23], [24], pointing out their use for regenerative medicine [23], [25]; Besides, similar reservoirs have been localized in lung and intestinal epithelium [26], [27]; however, these methods, mainly based on enzymatic protocols, yield mixed non-homogeneous populations or low numbers of cells from which it is extremely difficult to obtain single cell clones.

Here, we present a minimally invasive non-enzymatic method for isolating similar mesenchymal precursors from skin or lung adult tissues. Independently of the source, we found that a modification of the explant technique [14], [28] yielded high numbers of mesenchymal precursors in short time; moreover, populations from different sources were highly similar in surface and epithelial markers and were committed to differentiate into epithelium.

## Materials and Methods

### Isolation and in vitro Expansion of Mesenchymal Precursors

Mesenchymal precursor cells (MPCs) were isolated from adult tissues by the explant method [14]. Briefly, mice were sacrificed by cervical dislocation (Mice were maintained and used in accordance with the National Institutes of Health Animal Care, Spain, and the CNIC (Centro Nacional de Investigaciones Cardiovasculares) Institution approved their use.). Adult tissues were collected from 4-month-old C57BL6 mice and dissected into 1–2 mm pieces. The tissue fragments were examined under a microscope and those containing small blood vessels were selected. As a new approach, the tissue explants were placed in the centre of 24-plate wells coated with Matrigel (BDbiosciences, Franklin Lakes NJ, USA) around the well perimeter, and were orientated so as to expose blood vessels at the explant border. Explants were cultured in Dulbecco’s modified Eagles’s medium (DMEM, Sigma, St. Louis MO, USA) supplemented with 10% FBS (Sigma, St. Louis MO, USA)+Pen/Strep (Lonza, Basel, Switzerland)+L-glutamine (Lonza)+Hepes (Lonza, Basel, Switzerland), referred to as complete medium; culture was continued for several days in a humidified 5% CO2/95% air atmosphere at 37°C. After 10 days, a population of small, rounded and refractive cells floating above a fibroblast monolayer could be discerned and easily collected with a pipette. Clones were isolated by limited dilution and CFU-F colonies were formed (Three CFU-F colonies from each tissue explant of three different mice were obtained, amplified and characterized). The expansion of these cell populations was performed on gelatin-free culture plates in the same culture conditions. Derived clones were characterized by flow cytometry and gene expression pattern and routinely expanded over 20 passages. Cells were used for experiments between passages 5–10th. Explants were obtained under similar conditions from three different mice, and therefore, three independent adult mesenchymal precursor cell lines (MPCs) were obtained from each animal.

### Growth Curves

MPC clones maintained in 75 cm^3^ flasks were washed with PBS and detached by incubation in trypsin-EDTA solution for 30 seconds at 37°C. Cells were centrifuged for 5 min at 250 g at 4°C. Cells were plated in E-Plates 16 (Roche, Basel, Switzerland) at 255×10^3^ cells/cm^2^ (500000 cells/ml) in 100 µl complete medium, and incubated for at least 30 min in a culture hood at room temperature. Control wells only contained 100 µl cell-free complete medium. E-plates were inserted into the cradle pockets of the Real-Time Cell Analyzer (RTCA) DP Analyzer, and the background impedance of each well was measured. The number of proliferative cells was measured as Cell Index (CI), a dimensionless parameter derived as a relative change in measured electrical impedance to represent cell status. (CI is a quantitative measure of cell number present in a well. Additionally, change in a cell status, such as cell morphology, cell migration, or cell viability will lead to a change in CI). The RTCA DP Analyzer, placed in a standard cell-culture incubator, monitored cell number in each well every 15 min (quadruplicate measurements) over 48 h. MPCs showed a normal cell growth profile. Data were analyzed with the RTCA software (Roche, Basel, Switzerland).

### Flow Cytometry

Adult MPCs were detached from culture flasks with 5% EDTA on ice and incubated with pre-conjugated antibodies against surface markers for 25 min at 4°C. Surface markers analyzed were Sca1-PECy7, c-kit-PE, CD29-Fitc, CD34-PE, CD44-PECy5, CD45-v450, CD73-PE, CD90.2-Fitc and CD105-PE (BDbiosciences, Franklin Lakes NJ, USA). Cells were washed and labelled samples were analyzed in a BD FACS CantoII cytometer (BDbiosciences, Franklin Lakes NJ, USA). At least 10000 events were counted for each sample. All nine different MPCs clones were characterized by flow cytometry. Data analysis was performed with BD FACS DIVA (Table 1).

**Table 1:** Mean Fluorescence Intensity (MFI) of skin and lung MPCs surface markers.

	Skin		Lung	
%	MFI	Variance (+/−)	MFI	Variance (+/−)
**Sca-1**	29.4	1.2	25.2	2.3
**c-Kit**	35.1	3.4	31.1	1.4
**CD34**	37.7	2.1	34.2	3.7
**CD44**	62.3	2.2	56.1	3.8
**CD45**	0.8	0.3	0.6	0.2
**CD29**	16.5	0.2	18.3	1.1
**CD73**	27.2	4.1	21.2	1.4
**CD105**	33.2	1.6	29.7	1.3
**CD90.2**	35.9	3.3	39.6	2.3

Mean of nine independent experiments is shown (p<0.05 analyzed by t-Student Test).

### Reverse Transcription – Polymerase Chain Reaction

Total RNA was extracted from MPCs with Trizol reagent (TriReagent, Sigma, St. Louis MO, USA) and quantified spectrophotometrically (ND1000 Spectrophotometer, NanoDrop, Thermofisher Scientific, Whatham MA, USA). RNA integrity was monitored by ethidium bromide staining after agarose gel electrophoresis. RNA (1 µg) was reverse-transcribed to cDNA using the iScript cDNA Synthesis kit, with RNAase H+ and random hexamer primers included (BioRad, Hercules CA, USA). PCR was performed using a Veriti 96w thermal cycler (Applied Biosystems, Life Technologies, Paisley, UK) and PCR reaction mixtures contained cDNA templates, primers and 5′MasterMix (5Prime, Hamburg, Germany) in a final volume of 25 µl. Each PCR reaction was performed in triplicate, and each PCR also included no-template negative controls. Primers were designed using the Prime3 online program, checked with the Basic Local Alignment Search tool from NCBI, and made by Sigma (Sigma, St. Louis MO, USA. Primer concentration was optimized according to the PCR kit protocol. Embryonic primers were obtained from SABiosciences (Izasa, Spain). Primer sequences and amplification product lengths are shown in Table 2. Thermal cycling parameters were 94°C for 90 s; 35 cycles of 94°C for 45 s, the primer-specific annealing temperature for 45 s and elongation at 72°C for 60 s; and a final elongation at 72°C for 10 min. All PCR products were detected by ethidium bromide staining of 1.5% agarose gels.

**Table 2:** Primer sequences for RT-PCR.

	Primer Forward Sequence (5′-3′)	Primer Reverse Sequence (5′-3′)	(bp)*
*β-actin*	CACGATGGGAGGGGCCGGACTCAT	TAAAGACCTCTATGCCAACACAG	241
*GAPDH*	AATGCATCCTGCACCACCAA	GTGGCAGTGATGGCATGGAC	107
*Gata4*	CAGCATCTCTGTGGTCCTGA	GATGTTGTTGTGGCAAGTGG	259
*Nkx2.5*	CAGTGGAGCTGGACAAAGCC	TAGCGACGGTTCTGGAACCA	217
*c-actin*	GTGCCAGGATGTGTGACGA	CTGTCCCATACCCACCATGAC	153
*Cx43*	GGACTGCTTCCTCTCACGTC	TTTGGAGATCCGCAGTCTTT	204
*TropI*	CCACACGCCAAGAAAAAGTC	CGGCATAAGTCCTGAAGCTC	197
*Adipsin*	TCCGCCCCTGAACCCTACAA	TAATGGTGACTACCCCGTCA	319
*vWF*	TAATGGTGACTACCCCGTCA	GATGTTGTTGTGGCAAGTGG	217
*Kdr*	GGCGGTGGTGACAGTATCTT	GTCACTGACAGAGGCGATGA	162
*Tek*	AAGCATGCCCATCTGGTTAC	GTAGGTAGTGGCCACCCAGA	238
*Cdh5*	ATTGAGACAGACCCCAAACG	TTCTGGTTTTCTGGCAGCTT	239
*Pecam1*	TGCAGGAGTCCTTCTCCACT	ACGGTTTGATTCCACTTTGC	245
*Acta2*	CTGACAGAGGCACCACTGAA	CATCTCCAGAGTCCAGCACA	160
*Cdh1*	CAAGGACAGCCTTCTTTTCG	TGGACTTCAGCGTCACTTTG	165
*Nes*	CCAGAGCTGGACTGGAACTC	ACCTGCCTCTTTTGGTTCCT	161
*Hey1*	GAGACCATCGAGGTGGAAAA	AGCAGATCCCTGCTTCTCAA	210
*Gata2*	CCAGCAAATCCAAGAAGAGC	AGACTGGAGGAAGGGTGGAT	193
*Flt1*	TGAGGAGCTTTCACCGAACT	AGCTGGAGAAGCAGAAGCTG	206
*Endoglin*	CTTCCAAGGACAGCCAAGAG	GTGGTTGCCATTCAAGTGTG	221
*Esam*	TTGTTGGGTTGGTGCTGATA	GAGACACTGGGTGTGGGAGT	244
*Notch4*	AATGGGGGTACCTGTGTGAA	GTATAGCCAGGGCTGCAGAG	179
*Hmb45*	GCACCCAACTTGTTGTTCCT	GTGCTACCATGTGGCATTTG	160
*Idct*	AGCAGACGGAACACTGGACT	GCATCTGTGGAAGGGTTGTT	180
*Cdc42*	TTGTTGGTGATGGTGCTGTT	AATCCTCTTGCCCTGCAGTA	168
*Wnt3a*	ATGGCTCCTCTCGGATACCT	GGGCATGATCTCCACGTAGT	192
*End3*	GCTGGCAGAAAGACAGGAAC	CCGTTAAGCCAATCACCAGT	187
*Cadh2*	GGGACAGGAACACTGCAAAT	CGGTTGATGGTCCAGTTTCT	209
*Oct-4*	CTGTAGGGAGGGCTTCGGGCACTT	CTGAGGGCCAGGCAGGAGCACGG	198
*Nanog*	SasBiosciences	PPM25326C	145
*Sox2*	SasBiosciences	PPM04762E	160
*Cdx2*	SasBiosciences	PPM05771A	152
*Bmp2*	AACACTGTGCGCAGCTTCC	CTCCGGGTTGTTTTCCCAC	182
*Vimentin*	ACGAATACCGGAGACAGGTG	TCCAGCAGCTTCCTGTAGGT	143
*PDGFRb*	AAGCTCGGGTGACCATTCG	ACTTTCGGTGCTTGCCTTTG	219

* Amplicon length (bp).

### Western Blot

Adult MPCs were lysed directly with Laemmli buffer. Lysates were resolved by 10% SDS-PAGE. Proteins were transferred to nitrocellulose membrane (Hybond-ECL, GE Healthcare. NJ, USA). Membranes were probed with rabbit anti β-actin, rabbit anti α-tubulin and rabbit anti β1-integrin antibodies (AbCam, Cambridge, UK), and signal developed with enhanced chemiluminescence reagent (GE Healthcare, NJ, USA).

### Fluorescence Detection

Adult MPCs were grown on 0.1% gelatin-coated coverslips, washed with PBS and fixed with 4% paraformaldehyde for 15 min. Cells were maintained with donkey serum 1% for 1 h. Cells were incubated with each antibody for 1 h. For β actin detection, 1 µg/ml rabbit anti β-actin; for α-tubulin detection, 1 µg/ml rabbit anti α-tubulin; and for β1-integrin detection, 1∶500 rabbit anti β1-integrin (AbCam, USA). Cells were incubated with 1∶500 goat Alexa488 conjugated anti-rabbit secondary antibody. Cells were then stained with 300 nM DAPI for 30 min. Cells were maintained at room temperature during the process. Confocal images were obtained with a Leica DM2500– TSC.SPE microscope.

### Differentiation Protocols

To study epithelial differentiation, adult MPCs were plated on gelatin-free P6 culture plates (10^4^ cells/well) in MIX medium, composed of small airway basal medium (SABM, PromoCell, Heidelberg, Germany) supplemented with 10 µM *all-trans* retinoic acid (ATRA, Sigma, St. Louis MO, USA) and 50 µM keratinocyte growth factor (KGF, Sigma, St. Louis MO, USA). Control cells were incubated in complete medium. Cultures were maintained for 3 or 7 days before analysis. To induce adipogenic differentiation, MPCs were cultured in serum-free DMEM/F12 medium (1∶1) supplemented with 10 µg/ml transferrin, 15 mM NaHCO3, 15 mM HEPES, 33 µM biotin, 17 µM pantothenate, 1 nM insulin, 20 pM triiodothyronine, 1 µM cortisol, plus antibiotics. Accumulation of triglycerides in adipocytes was visualized by staining formalin-fixed cells with Oil Red O. In order to measure the osteogenic potential of the MPCs, 2×10^4^ cells were incubated in complete expansion medium until a confluent layer was achieved and then osteogenic medium was added, containing IMDM supplemented with 9% FBS, 9% HS, 2 mM L-glutamine, 100 U/mL penicillin, 100 µg/mL streptomycin, 50 ng/mL L-thyroxine, 20 mM β-glycerol phosphate, 100 nM dexamethasone, and 50 µM ascorbic acid. Medium was refreshed every 3–4 d. After 15 d of culture cells were fixed in 10% formalin and stained with 10% Alizarin Red. To measure the chondrogenic differentiation of ASCs, 5×10^5^ cells were incubated in 500 µL of complete chondrogenic medium (CCM), containing IMDM, 100 nM dexamethasone, 50 µg/mL ascorbic acid, 40 µg/mL L-proline with ITS + supplement, 1 mM sodium pyruvate, 100 U/mL penicillin, 100 µg/mL streptomycin, 10 ng/mL TGFβ-3 and 100 ng/mL BMP-2. The medium was changed three times a week. After 18 d, cells were stained with Toluidine Blue sodium borate. Finally, differentiation into smooth muscle cells was induced by treatment with TGFβ1 and BMP-4, while co-culturing MPCs with C2C12 myoblasts induced differentiation into skeletal muscle cells. All experiments were done with three different MPCs clones and results are expressed as percentage of differentiation capacity for each treatment.

### Cell Migration

The upper chamber wells of CIM-Plates 16 (Roche, Basel, Switzerland) were precoated with fibronectin, added to wells at 10 µg/ml (30 µl) and removed after 30 min. The lower chamber wells were filled with 160 µl culture medium, allowing a clearly-defined meniscus to form. SDF1, MCP1 and TNFα (Peprotech Inc. Rochy Hill, NJ, USA) were added to the lower chambers at a final concentration of 100 ng/ml. CIM-Plates were equilibrated for at least 30 min in a culture hood at room temperature. Trypsin-EDTA-dispersed MPCs (100 µl in complete medium) were then plated in the fibronectin-precoated upper chamber wells at 306×10^3^ cells/cm^2^ (600000 cells/ml) in 100 µl complete medium, which we determined as the optimal density to ensure confluent monolayers. The CIM-Plates were inserted into the cradle pockets of the RTCA DP Analyzer, and the background impedance of each well was measured after 1 h. The RTCA DP Analyzer, placed in a standard cell-culture incubator, monitored the migrated cell number in each lower chamber well every 15 min (quadruplicate measurements) over 36 h. Data were analyzed with the RTCA Software.

### Statistical Analysis

All data are represented as mean ± SEM from 3–9 independent experiments. Comparisons between two groups were done by Student’s *t* test. One-way or two-way ANOVA were used as required by the assay. Differences between groups were considered statistically significant at *P*<0.05.

## Results

### Isolation of Mesenchymal Precursor Cells from Different Adult Tissues

**Figure 1:**
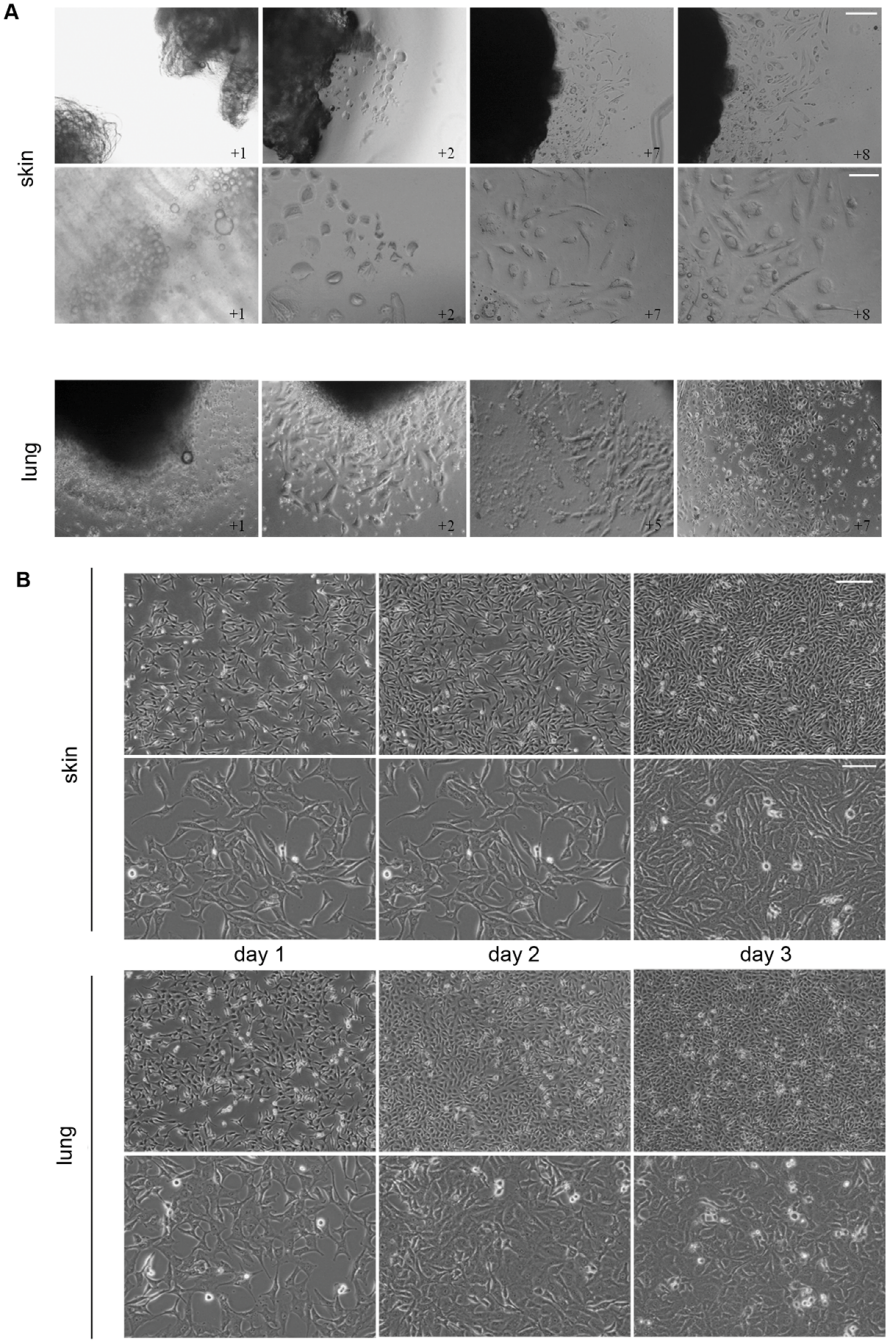
Mesenchymal precursors isolated from adult mouse tissue. A. Representative phase contrast images of one explant out of three independent explant assays performed with three different animal tissues are shown. To note outgrowth of mesenchymal precursor cells (MPCs) already at day 2 from skin and lung tissue explants surrounded by a ring of Matrigel. Images were taken 1, 2, 7 and 8 days after explant attachment. Scale bars, 100 µm (explants) and 30 µm (cultures). B. Growth and morphology of MPC cultures. Skin and lung MPCs obtained from explants were maintained in culture, and representative phase contrast images taken 1, 2 and 3 days after plating. Scale bars, 100 µm (upper panel) and 30 µm (lower panel).

Mesenchymal precursors from different adult tissues were isolated using the explant method [14]. Several modifications were added in order to standardize the protocol and therefore enable isolation of similar mesenchymal precursor populations from most adult tissues. Biopsies of lung and skin (Figure 1A), together with skeletal muscle, heart and adipose tissue as controls (data not shown), were collected from 4-month-old C57BL6 mice, washed in PBS and dissected into 1–2 mm pieces with a scalpel. 24-well plates were prepared by coating the well perimeters with a thick layer of Matrigel, leaving the centre clear to allow attachment of the explant. The dissected tissue fragments were examined under a microscope and those containing small blood vessels were selected and placed in the centre of the tissue plate wells. Explants were cultured in complete medium under standard conditions (see Materials and Methods). In skin explant cultures lipid drops appeared during the first 2 days, and after 2 days fibroblasts started to grow out from the explant toward the Matrigel. After a further 3 days, abundant small round, refractive cells emerged, and migrated above the fibroblast monolayer toward the Matrigel border (Figure 1A). The round, refractive cells continued to emerge up to day 14 after plating the explant (Figure 1A). A similar process was observed in lung explant cultures, with a more rapid outgrowth than skin explants (Figure 1A). The small round, refractive cells were collected from lung explant cultures after 14–20 days. The collected cells were cloned by limited dilution culture on gelatin-free culture plates in complete medium and CFU-F colonies were formed. Clones were putatively identified as mesenchymal precursor cells (MPCs) based on cell colony morphology and surface marker expression (see below). Explants were obtained under the same conditions from three different mice, and three independent adult mesenchymal precursor lines (MPCs) were obtained from each animal. Skin and lung MPCs had similar cell morphology during culture expansion (Figure 1B); however, at confluence, skin MPCs had a more elongated shape, whereas lung MPCs had a more polygonal form (Figure 1B).

Similar outcomes were obtained with explants from the other control adult tissues (data not shown), and the mesenchymal cells obtained from cardiac, skeletal muscle and adipose tissue had similar properties to those described in previous studies [15], [28], [29]. Here, we present a minimally invasive non-enzymatic method for isolating similar mesenchymal precursors from skin or lung adult tissues. Independently of the source, we found that a modification of the explant technique [14], [28] yielded higher numbers of mesenchymal precursors in short time.

### Determination of MPC Proliferation Index

**Figure 2:**
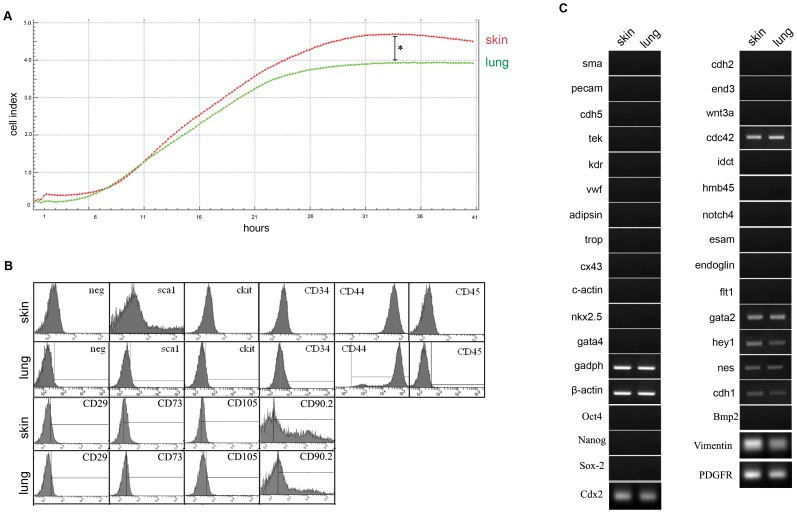
Characterization of skin and lung MPCs. A. Representative growth curves for skin (red) and lung (green) MPCs in complete medium. One out of nine wells of representative cultures in a Real Time Cell Analyzer is shown. Cell Index represents the relative number of proliferative cells. Differences between skin and lung were statistically significant by ANOVA (*p<0.03). B. Flow cytometry analysis of surface molecule expression on skin and lung mesenchymal precursors. One group of representative histograms out of nine independent experiments is shown. Table 1 shows the media of the mean intensity fluorescence and the variance for all nine experiments. C. RT-PCR analysis of gene expression profile in skin and lung MPCs. The panel shows one representative agarose gel analysis of amplified products out of three independent experiments for each explant. Primer sequences are shown in Table 2 and summary of the results is shown in Table 3.

Proliferation growth curves of skin and lung MPCs were determined with the Real Time Cell Analyzer from Roche. Nine different skin or lung MPCs clones were plated at 5×10^4^/well in complete medium, and cells were counted automatically every 15 min over 48 hours for each well (Figure 2A; one representative well). The number of proliferative cells was measured as Cell Index. The CI mean values of both MPC populations were similar, with skin MPCs reaching a plateau after 33 h compared with 31 h for lung MPCs. However, the total cell number in confluent cultures was significantly higher for skin MPCs (CI: 4.7+/−0.2), indicating an overall higher proliferation capacity than lung precursors (CI: 3.9+/−1).

### Characterization of Surface and Gene Markers

Surface markers on nine different skin and lung MPCs clones were analyzed by flow cytometry (Figure 2B). Skin and lung clones were positive for stem cells markers such as Sca1 and c-Kit and also positive for mesenchymal surface markers such as CD34, CD44, CD29, CD73, CD105 and CD90.2. In contrast, both clones were negative for the hematopoietic maker CD45 (Figure 2B and Table 1). Surface marker expression was not affected by long-term culture (up to passage 20) in either MPC population (data not shown).

The MPC clones were further analyzed for gene expression by RT-PCR (see primers in Table 2), revealing a similar gene expression pattern in skin and lung precursors (Table 3). Both cell populations expressed the constitutive markers β-actin and GADPH, as well as the multipotency markers cdc42 and nestin, which are widely expressed markers in skin and lung tissue (Figure 2C and Table 3). Nevertheless, both populations were negative for the embryonic pluripotent markers Oct4, Nanog and Sox2, while positive for Cdx2. Both MPCs populations expressed the mesenchymal lineage vimentin and PDGFb, being only negative for the mesenchymal marker Bmp2 (Figure 2C and Table 3). They also expressed the epithelial markers cdh1, hey1 and gata2, but were negative for the endothelial markers vWF, kdr, tek, cdh5, flt1, endoglin, esam and notch4 and for α-smooth-muscle actin. Similarly, neither population expressed the cardiac markers gata4, nkx2.5, c-actin, cx43 and tropI, the adipocyte marker adipsin, and the neural marker cdh2 (Figure 2C and Table 3). Both populations were also negative for the skin-specific markers hmb45, idct, wnt3a and end3. In all cases gene expression was not affected by passage. This isolation protocol thus yields cell populations from different adult tissues with similar surface marker and gene expression profiles.

**Table 3:** Gene expression profile of skin and lung MPCs.

Marker	Gene	Skin MPCsexpression	Lung MPCsexpression
constitutive	β-actin	+	+
constitutive	gadph	+	+
cardiac	gata4	−	−
cardiac	nkx2.5	−	−
cardiac	c-actin	−	−
cardiac	cx43	−	−
cardiac	tropI	−	−
adipocytic	adipsin	−	−
endothelial	vwf	−	−
endothelial	kdr	−	−
endothelial	tek	−	−
endothelial	cdh5	−	−
endothelial	flt1	−	−
endothelial	endoglin	−	−
endothelial	esam	−	−
endothelial	notch4	−	−
smooth muscle	α-sma	−	−
neural	nes	+	+
neural	cdh2	−	−
epithelial	cdh1	+	+
epithelial	hey1	+	+
epithelial	gata2	+	+
melanocytes	hmb45	−	−
melanocytes	idct	−	−
skin	cdc42	+	+
skin	wnt3a	−	−
skin	end3	−	−
embryonic	Oct4	−	−
embryonic	Nanog	−	−
embryonic	Sox2	−	−
embryonic	Cdx2	+	+
mesenchymal	Bmp2	−	−
mesenchymal	Vimentin	+	+
mesenchymal	PDGFb	+	+

The table summarizes the results of RT-PCR analysis shown in Figure 2C (3 independent MPCs clones analyzed from each explant).

### Cytoskeletal Structure

**Figure 3:**
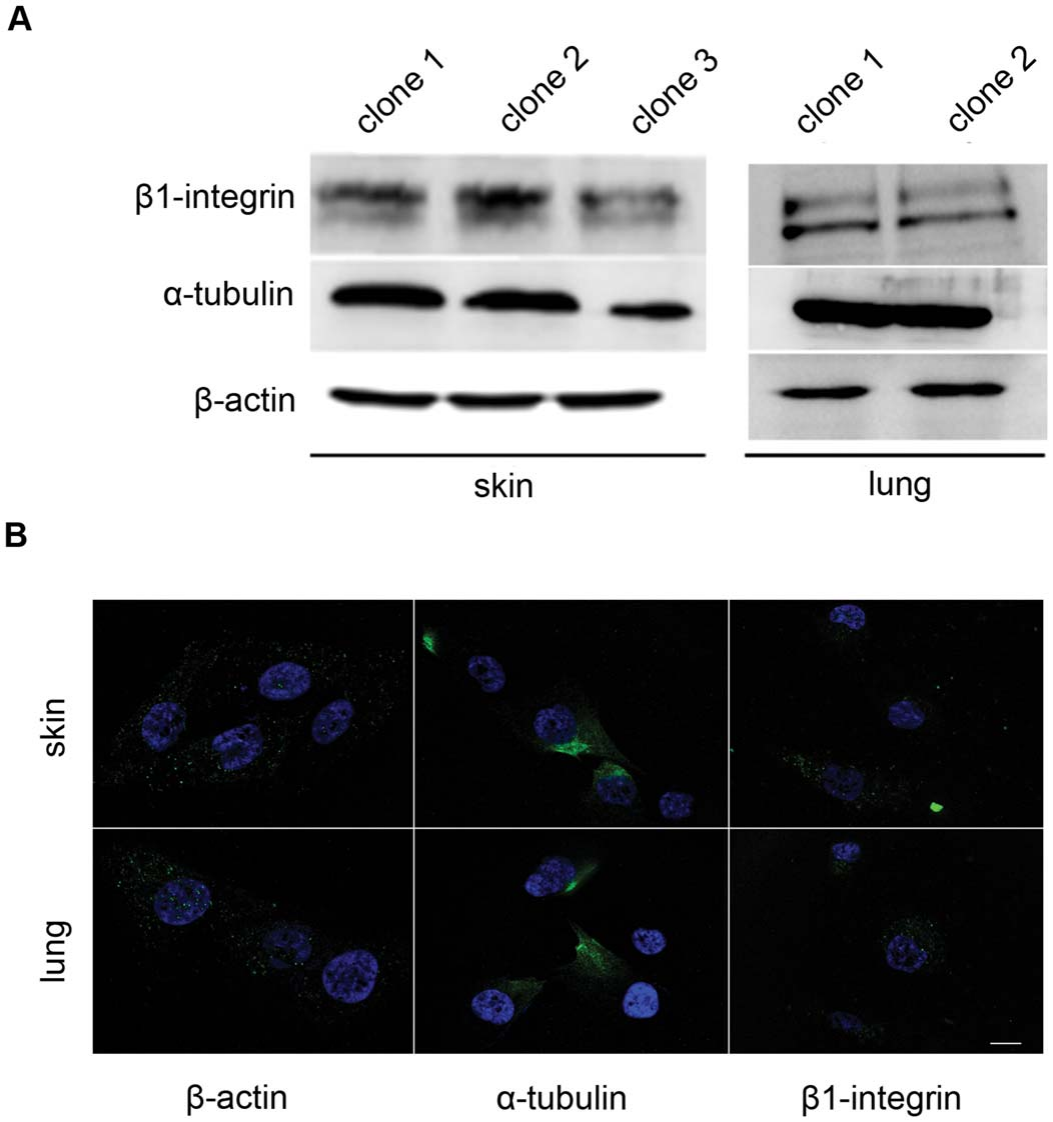
Cytoskeletal protein expression and localization in MPCs. A. Western blot showing constitutive expression of cytoskeletal components in mesenchymal precursors from skin and lung. One out of three independent experiments is shown. B. Representative fluorescence images of skin and lung MPCs, showing the distribution of α-actin, β-tubulin and β1-integrin rabbit anti-mouse antibody). Scale bar, 1 µM.

The constitutive gene expression of cytoskeletal components suggests viability of both MPC populations. To confirm that isolation and expansion did not disturb the correct orientation of cytoskeletal proteins, we analyzed cell structure in three independent skin and lung MPCs clones. Western blot analysis confirmed that both populations express similar levels of β-actin, α-tubulin and β1-integrin proteins (Figure 3A). Immunofluorescence confirmed that skin and lung precursor clones have a similar distribution of β-actin, α-tubulin and β1-integrin (Figure 3B). Moreover, the distribution of cytoskeletal proteins was not modified by passage.

### Differentiation Properties of Skin and Lung Mesenchymal Precursors

**Figure 4:**
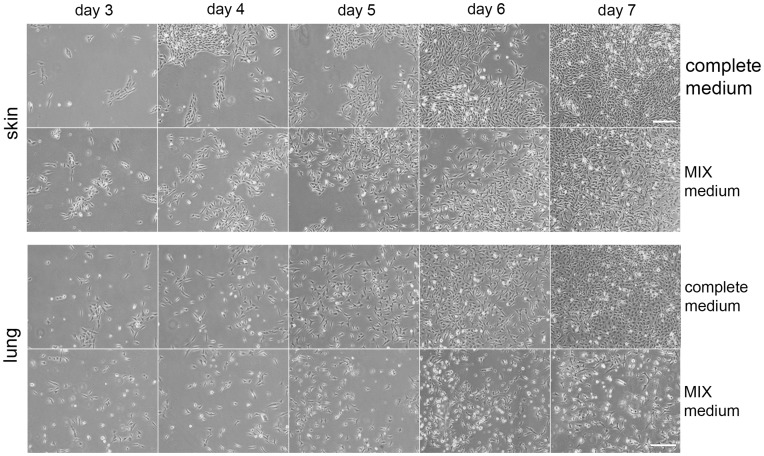
MPC differentiation. Skin and lung MPCs were grown in complete medium or in epithelial differentiation medium (MIX medium) over 7 days. Images were taken along the seven days of differentiation. To note that cells grown in MIX medium acquired epithelial morphology, consistent with gene expression changes (Table 4). Three independent experiments were performed. Scale Bar, 100 µm.

The differentiation capacity of MPCs to generate epithelium was tested by incubation of three independent MPCs clones in a specifically tailored medium (MIX medium), containing ATRA, KGF and SABM (details in Materials and Methods). Over the 7 days treatment period, cells of both origins became more elongated and slowed proliferation (Figure 4). Gene expression analysis by RT-PCR on days 3 and 7 showed that skin and lung MPCs cultures both reduced the expression of the multipotent marker nestin and started expressing Esam and vWF, markers for epithelial differentiation (Table 4). After 7 days nestin expression had disappeared, while the expression of Esam and vWF was further accompanied by kdr expression (Table 4). These results suggest that MPCs could differentiate into mature epithelium after stimulation. To determine the potential ability of MPCs to differentiate into other lineages, specific differentiation mediums to induce adipose, osteoblast, chondroblast, smooth muscle and skeletal muscle differentiations were added to the cultures (see materials & methods for details). As shown in Figure S1, both skin and lung MPCs clones could only differentiate into epithelium and were not able to generate adipocytes, osteoblasts, chondroblasts or smooth or skeletal muscle fibers in a significant manner.

**Table 4:** Changes on lineage marker expression of MPCs cultured for 7 days in epithelial differentiation medium (MIX medium) compared with growth complete medium.

Marker	Gene	Skin	Lung	Skin	Lung
		Complete Medium	Complete Medium	MIX Medium	MIX Medium
constitutive	β-actin	+	+	+	+
constitutive	Gadph	+	+	+	+
cardiac	gata4	−	−	−	−
cardiac	nkx2.5	−	−	−	−
cardiac	c-actin	−	−	−	−
cardiac	cx43	−	−	−	−
cardiac	tropI	−	−	−	−
adipocytic	adipsin	−	−	−	−
endothelial	**vwf**	−	−	**+**	**+**
endothelial	**kdr**	−	−	**+**	**+**
endothelial	tek	−	−	−	−
endothelial	cdh5	−	−	−	−
endothelial	flt1	−	−	−	−
endothelial	endoglin	−	−	−	−
endothelial	**esam**	−	−	**+**	**+**
endothelial	notch4	−	−	−	−
smooth muscle	α-sma	−	−	−	−
neural	**nes**	**+**	**+**	−	−
neural	cdh2	−	−	−	−
epithelial	cdh1	+	+	−	−
epithelial	hey1	+	+	+	+
epithelial	gata2	+	+	+	+
melanocytes	hmb45	−	−	−	−
melanocytes	idct	−	−	−	−
skin	cdc42	+	+	+	+
skin	wnt3a	−	−	−	−
skin	end3	−	−	−	−

The table summarizes the results of RT-PCR analysis shown in Figure 4 (3 independent MPCs clones analyzed from each explant).

### Cell Migration Capacity

**Figure 5:**
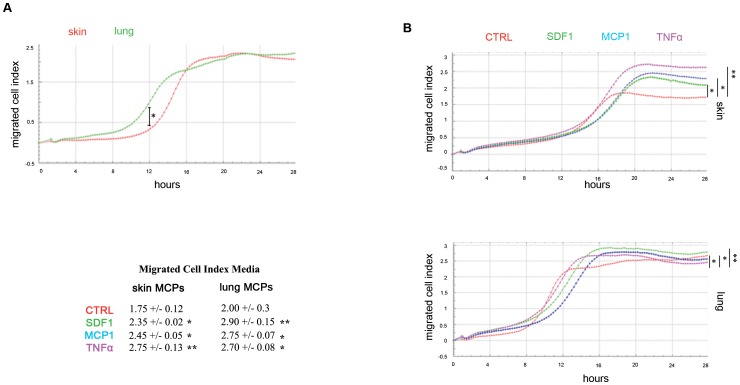
Mesenchymal precursor migration. A. Migration of undifferentiated skin (red) and lung (green) MPCs analyzed by a well insert assay in the Real Time Cell Analyzer. One representative experiment out of 9 independent wells of MPCs cultures is shown. Migrated Cell index is the relative number of migrated cells. Differences between skin and lung at 12 h were statistically significant by ANOVA (*p<0.04). B. Migration of skin and lung MPCs in response to cytokines. The indicated stimuli were placed in the lower chambers in the migration assay. One representative experiment out of 9 independent wells of lung and skin MPCs cultures in presence of cytokines is shown. Data were analyzed as in A. Differences between control and stimulated cells were statistically significant by ANOVA (*p<0.05; **p<0.04). Table with the mean of migrated cell index for the nine control or stimulated MPCs clones is shown. CTRL, un-stimulated control cultures.

The migration capacity of undifferentiated skin and lung mesenchymal precursors was examined by a well insert assay. Nine independent skin and lung MPCs clones were plated in the upper chamber and migration to the lower chamber was monitored every 15 min over 36 hours by the Real Time Cell Analyzer. The number of migrated cells was measured as migrated Cell Index (Figure 5A). Skin and lung MPCs showed a similar migration capacity in the absence of specific stimuli, with a slightly higher number of migrated cells in skin precursor cultures (Figure 5A). Skin MPCs began to migrate 12 h after seeding, and achieved a migrated CI of 1.75+/−0.12 after 5 h. Lung MPCs began to migrate earlier, at 9 h post seeding, and reached a migrated CI of 1.6+/−0.3 after 7 h. Thus skin mesenchymal precursor migration began 3 h later but achieved a higher migrated cell index in less time (Figure 5A).

The migratory response of MPCs to stimuli was tested by adding SDF1, MCP1 or TNFα to the lower chamber; all these factors have been described as signals that induce mesenchymal cell migration [28], [30]. Skin and lung MPCs both showed an enhanced migration capacity in response to these stimuli; however, the migration responses of skin and lung precursors differed (Figure 5B). The migrated CI for skin MPCs was 2.35+/−0.02 in response to SDF1 (green), 2.45+/−0.05 to MCP1 (blue) and 2.75+/−0.13 to TNFα (pink), whereas the corresponding CI for lung MPCs were 2.90+/−0.15, 2.75+/−0.07 and 2.70+/−0.08 (Figure 5B). Thus, skin MPCs were more responsive to TNFα, followed by MCP1 and SDF1, whereas lung MPCs were more responsive to SDF1, followed by MCP1 and TNFα. The migratory capacity of MPCs suggests potential to reach areas of damage in cell therapy.

## Discussion

In this study we establish a simple, reproducible and rapid method for the isolation of pre-committed adult mesenchymal precursors from a variety of sources, including tissues that are more easily accessible such as skin. The explant method has been previously employed for the isolation of specific populations such skeletal or cardiac mesoangioblasts as well as for obtaining cardiospheres cultures [14], [28], [29]. Nevertheless, here we slightly modify the technique by adding an extracellular matrix rich in factors that attract the mesenchymal precursors and apart from increasing the quantity and the timing for obtaining these cell populations, allow us to process any adult tissue in a similar manner. The method is extremely easy and efficient for isolating this kind of mesenchymal precursor from any given tissue and, given the advantages of skin as an accessible tissue source, our protocol therefore has great potential for the future development of autologous transplantation procedures for humans. Our current findings describe the first isolation of similar pre-committed mesenchymal precursors from mammalian skin and lung. *Toma* et al. reported evidence that mesenchymal precursors might reside in adult mammalian skin [23]. Subsequently, it was demonstrated that small punch biopsies of human skin contained multipotent mesenchymal cells that proliferated as spheroid aggregates in response to FGF2 and EGF [24], [31], [32]. More recently, different multipotent progenitors have been described to generate all cell lineages of the skin and can be identified with specific markers such as Lrig1 and Lgr6, which are negative for our MPCs population [20], [22]. In contrast, the mesenchymal precursors described here are able to proliferate without stimuli and grow as non-aggregated adherent cells, showing an elongated morphology, are already committed to one lineage, epithelium, and are negative for pluripotent markers. In the case of the lung, previous protocols for isolating multipotent cells from lung require aggressive enzymatic methods or expansion on feeder layers in conditioned medium [26], [33], [34]. With the modified explant method used here, committed mesenchymal precursors from lung or skin are isolated without enzymatic or mechanical processing and require no feeder layers or special medium for expansion, and generate useful numbers of functional cells in less than two weeks.

Skin and lung MPCs differed in the timing of the onset of proliferation, but reached a similar rate after a couple of weeks. The initial difference might be due to the greater vascularization of lung compared with skin, implying a greater density of precursors in lung explants [35].

The surface marker expression profiles of skin and lung MPCs suggest that these cell lines have self-renewal potential, through their expression of stem cells markers such as c-kit and sca1. Both populations also express typical mesenchymal markers such as CD29, CD73, CD105, CD90.2, CD44 and CD34, which has been reported at variable levels on murine mesenchymal stem cells [36], [37]. Moreover, neither MPC population expressed the hematopoietic surface marker CD45. A similar mesenchymal expression profile is detected in cells cultured of mesenchymal precursors isolated by a similar method from heart, skeletal muscle or adipose tissue [15], [28], [29].

Despite their different embryonic origin, skin and lung MPCs also have a highly similar expression profile of lineage genes. Apart from expressing typical mesenchymal genes such as vimentin and PDGFb, both populations express the well-known skin epithelial marker cdh1, as well as the epithelial markers hey1 and gata2, suggesting that these cells are already pre-committed to epithelial lineages. In contrast, neither population expresses the embryonic markers Oct4, Nanog or Sox2, indicating their lack of pluripotency [38]; neither MPCs line expresses the specific skin markers hmb45, idct, wnt3a or end3, although both express the skin-related marker cdc42, which is also expressed in other epithelial lineages. While skin and lung MPC lines were negative for the neural marker cdh2, both populations were positive for nestin; this intermediate filament, in addition to being a neural marker [39], is also a marker of stemness expressed widely during development and in several types of adult stem cell [40], [41], [42]. The skin and lung MPCs were also negative for cardiac, endothelial, adipose and smooth muscle lineage markers. Therefore, skin and lung MPCs appear to represent a homogeneous population of mesenchymal precursors pre-committed to the differentiation of epithelial lineages.

Treatment with differentiation medium (MIX medium) confirmed the capacity of MPCs to differentiate further toward an epithelial fate. MPCs switch off nestin expression after 3 days of treatment. This timing is consistent with the observed induction of epithelial genes in embryonic bodies [41]. Differentiating MPCs also express the epithelial marker Esam and the endothelial markers vWF and kdr, suggesting that mesenchymal precursors from adult skin and lung differentiate to specific cell types related to epithelial and endothelial function. MPCs thus might constitute an adult precursor stock for the replacement of specific cell types. The initial proliferation advantage of lung MPCs during expansion from explants might reflect the greater blood vessel density in lung tissue [35]. In subsequent culture, this advantage is reversed, with skin MPCs reaching higher overall numbers in proliferation assays. This might reflect the high renewal rate of skin, which requires a higher proliferation capacity [42].

Effective cell therapy strategies require the implanted cells to be able to migrate to sites of damage, in order to avoid invasive delivery procedures. Skin and lung MPCs both showed an ability to migrate under basal conditions and in response to SDF1, MCP1 and TNFα, described as signals that induce migration of different mesenchymal cells [30]. Skin MPCs migrated faster than lung MPCs. This might also be related to the lower blood vessel density in skin, requiring skin MPCs to migrate further to reach their destination [35]. The differential response of lung and skin MPCs to SDF1, MCP1 and TNFα, with lung cells responding more potently to SDF1 and skin cells responding to TNFα, indicate that despite the overall similar functionality of the two populations they show specific responses related to their tissue of origin. This differential potential will need to be taken into account in the development of clinical protocols with this kind of precursor.

### Conclusions

This study presents a modified explant technique method for isolating homogeneous committed mesenchymal precursor cells from skin and lung. This MPCs population express mesenchymal markers and show pre-commitment to epithelial lineages. The ease of obtaining and expanding these cells, especially from skin, together with their migratory and differentiation capacity, mark them as attractive candidates for translation to clinical strategies employing autologous cells for regenerative therapy. The similar gene and surface marker expression in skin and lung MPCs highlights that similar kinds of precursors can be isolated from different tissues using a simple and standardized procedure. Nonetheless, the specific responses of skin and lung MPCs to migratory stimuli suggests that precursors derived from a specific tissue might not be suitable for the therapeutic replacement of epithelial cells in other tissues.

## Supporting Information

Figure S1 **Differentiation properties of MPCs.** Percentages of skin and lung MPCs clones that differentiate into adipose, chondrogenic, osteogenic or muscle tissues in three independent experiments (Student T-test; *p<0.01).
(TIF)
