# Supplementary figures and images for: Method for Obtaining Committed Adult Mesenchymal Precursors from Skin and Lung Tissue
Source: PLoS One. 2012 Dec 31;7(12):e53215. doi: 10.1371/journal.pone.0053215 (PMC3534150; doi:10.1371/journal.pone.0053215)

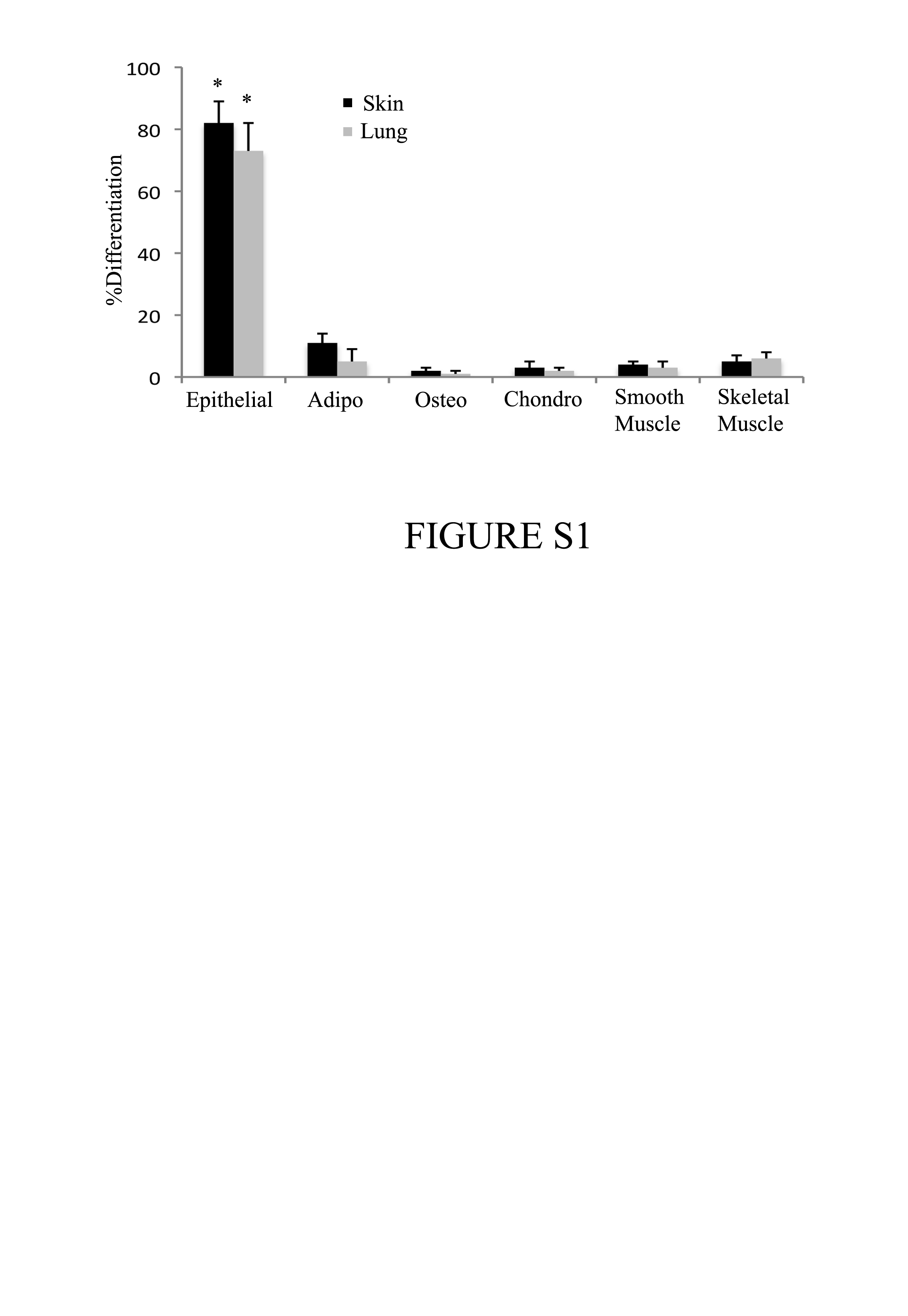

Supplement: Figure S1 — Differentiation properties of MPCs. Percentages of skin and lung MPCs clones that differentiate into adipose, chondrogenic, osteogenic or muscle tissues in three independent experiments (Student T-test; *p<0.01). (TIF) [file pone.0053215.s001.tif]
